# Fronto‐parieto‐subthalamic activity decodes motor status in Parkinson's disease

**DOI:** 10.1111/cns.14155

**Published:** 2023-04-05

**Authors:** Quan Zhang, Hutao Xie, Baotian Zhao, Zixiao Yin, Yuye Liu, Defeng Liu, Yutong Bai, Guanyu Zhu, Guofan Qin, Yifei Gan, Runfa Tian, Lin Shi, Anchao Yang, Fangang Meng, Yin Jiang, Jianguo Zhang

**Affiliations:** ^1^ Department of Neurosurgery Beijing Tiantan Hospital, Capital Medical University Beijing China; ^2^ Department of Functional Neurosurgery, Beijing Neurosurgical Institute Capital Medical University Beijing China; ^3^ Beijing Key Laboratory of Neurostimulation Beijing China

**Keywords:** local field potentials, machine learning, motor decoding, parietal lobule, Parkinson's disease

## Abstract

**Aims:**

Patients with Parkinson's disease (PD) have various motor difficulties, including standing up, gait initiation and freezing of gait. These abnormalities are associated with cortico‐subthalamic dysfunction. We aimed to reveal the characteristics of cortico‐subthalamic activity in PD patients during different motor statuses.

**Methods:**

Potentials were recorded in the superior parietal lobule (SPL), the primary motor cortex (M1), premotor cortex (PMC), and the bilateral subthalamic nucleus (STN) in 18 freely walking patients while sitting, standing, walking, dual‐task walking, and freezing in medication “off” (Moff) and “on” (Mon) states. Different motor status activities were compared in band power, and a machine learning classifier was used to differentiate the motor statuses.

**Results:**

SPL beta power was specifically inhibited from standing to walking, and negatively correlated with walking speed; M1 beta power reflected the degree of rigidity and was reversed by medication; XGBoost algorithm classified the five motor statuses with acceptable accuracy (68.77% in Moff, 60.58% in Mon). SPL beta power ranked highest in feature importance in both Moff and Mon states.

**Conclusion:**

SPL beta power plays an essential role in walking status classification and could be a physiological biomarker for walking speed, which would aid the development of adaptive DBS.

## Graphical abstract

This study showed SPL beta power indicated the physiological walking activities both in the medication “off” and “on” states, and beta power in M1 reflected the degree of rigidity and could be reserved by medication. These findings provide evidence for decoding PD patients' movements and sheds new insight into the development of intelligent closed‐loop DBS.

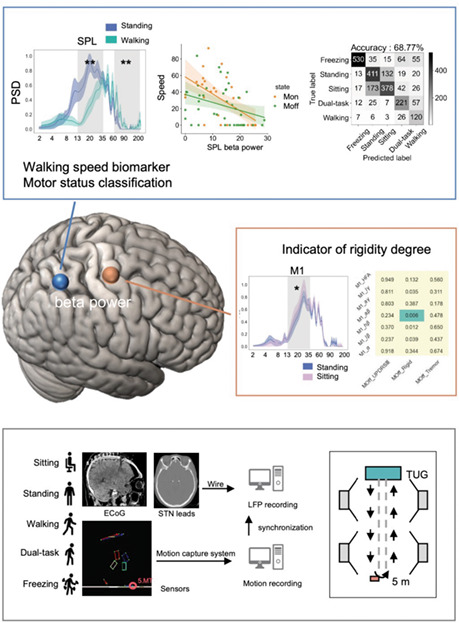

## 1 INTRODUCTION

Eighty percent of patients with Parkinson's disease (PD) demonstrate dysfunctions in various motor status switches, such as difficulties in standing up, gait initiation, and freezing while walking.^1^ These dysfunctions are the first intrinsic causes associated with falls and are closely related to cognitive impairment, often leading to restrictions in the patient's quality of life.^2^, ^3^, ^4^ In addition, with disease progression, these dysfunctions are resistant to dopaminergic treatment and deep brain stimulation (DBS).^5^, ^6^ Therefore, revealing the neurophysiological patterns underlying different motor statuses, including sitting, standing, walking, dual‐task walking, and freezing of gait, is essential for developing the optimized therapy.

Previous studies suggested that the degeneration of multiple subcortical/cortical regions is involved in motor dysfunction. In PD patients, increased gray matter atrophy in the primary motor cortex (M1) is associated with more severe gait disorder,^7^ and the STN oscillations correlate with vulnerability to freezing in PD patients.^8^ The superior parietal cortex (SPL) and premotor cortex (PMC) are components of the fronto‐parietal network, whose activities are impaired during gait dysfunction.^9^, ^10^ In addition, SPL and PMC are crucial elements for the coordination of the lower limb muscles. They are involved in adapting to locomotor speed on the treadmill and are essential for stimulus‐driven action.^11^, ^12^ Nevertheless, the specific characteristics of these brain regions during different motor statuses in PD patients remain unclear.

We hypothesize that there are characteristic frontal‐parieto‐subthalamic activities that occur during different motor statuses in PD patients. Therefore, we utilized electrocorticography (ECoG) of the SPL, M1, and PMC, and acquired simultaneous bilateral STN local field potentials in freely walking PD patients (*n* = 18). Those patients performed five motor statuses (sitting/standing/walking/dual‐task/freezing) both in the medication “off” and medication “on” states. We used conventional univariate analyses to identify the critical features that distinguish motor statuses in the frequency domain during the medication “off” state, then employed a machine learning classifier to differentiate the motor statuses and verify the findings during the medication “on” state.

## 2 MATERIALS AND METHODS

### 2.1 Human Subjects

Eighteen patients with PD were recruited for the study. Overall, the subjects had an average age of 65.94 years, with an average disease duration of 9.50 years. The average pre‐operative Unified Parkinson's Disease Rating Scale part III (UPDRS III) scores were 48.56 in the medication “off” state and 21.11 in the medication “on” state. All participants provided written informed consent to participate, which was approved by the Institutional Review Board of Beijing Tiantan Hospital. The subjects' preoperative assessments can be found in our previous reports.^13^, ^14^, ^15^ The participants' demographic and clinical information is summarized in Table 1.

**TABLE 1:** Demographics of the subjects.

ID	Age/Sex	DD (years)	LEDD (mg)	UPDRSIII		Rigid		Tremor		FOGQ	State^g^
				Moff^a^	Mon^b^	Moff^c^	Mon^d^	Moff^e^	Mon^f^		
sub01	72/M	10	675	47	28	7	3	17	11	10	off/on
sub02	60/F	7	750	47	24	8	3	13	3	20	off
sub03	57/F	5	375	49	24	8	1	10	0	14	off
sub04	66/F	10	513	61	22	5	2	11	4	21	off/on
sub05	53/M	12	1100	79	25	14	5	18	4	24	off
sub06	70/M	12	688	70	37	9	7	20	11	17	on/off
sub07	73/F	9	1439	51	27	9	5	0	0	20	off/on
sub08	67/F	6	500	52	30	9	5	11	3	22	off/on
sub09	59/F	9	700	46	21	10	5	6	3	16	on/off
sub10	78/M	5	550	58	24	12	6	5	3	18	off
sub11	76/M	8	1351	41	11	4	2	3	3	13	off/on
sub12	66/F	15	669	55	8	10	3	11	1	13	on/off
sub13	61/M	7	1150	37	18	10	5	0	0	22	on/off
sub14	67/M	10	913	42	20	10	1	2	4	16	on/off
sub15	66/F	15	925	39	20	7	3	1	0	20	off/on
sub16	67/F	9	1000	27	5	2	1	0	0	15	off/on
sub17	71/F	11	1212	36	19	6	3	6	3	19	on/off
sub18	58/M	11	1263	37	18	8	4	0	0	9	on/off

Abbreviations: DD, disease duration; FOGQ, freezing of gait questionnaire; LEDD, levodopa equivalent daily dose.

^a^ MDS‐UPDRS III off‐medication score.

^b^ MDS‐UPDRS III on‐medication score.

^c^ MDS‐UPDRS III off‐medication rigidity score, including 3.3.

^d^ MDS‐UPDRS III on‐medication rigidity score, including 3.3.

^e^ MDS‐UPDRS III off‐medication tremor score, including 3.15, 3.16, 3.17, and 3.18.

^f^ MDS‐UPDRS III on‐medication tremor score, including 3.15, 3.16, 3.17, and 3.18.

^g^ Medication state order in the study. off: Medication “off” state; on: Medication “on” state.

### 2.2 STN and subdural electrodes implantation

All subjects underwent implantation of DBS leads (PINS L301 PINS, Beijing, China) in the bilateral sensorimotor region of the STN and subdural strip electrodes (Sinovation, Beijing, China) in the right hemisphere. The eight‐contact subdural strip electrodes, whose diameters were 8 mm with an exposure of 80 mm and an intercontact distance of 10 mm (two subjects with 10‐contact strip electrodes, and one subject with 30‐contact strip electrodes arranged in two rows with 1.5 mm diameters, 80 mm exposure, and 5 mm intercontact distance), were placed through the DBS burr hole, and parallel to the direction of the superior sagittal sinus, with contacts spanning the PMC, M1, S1, and SPL (shown in Figure S1). The electrode extension cables were externalized through the scalp for electrophysiological recordings. The positions of the electrodes were confirmed using thin‐slice CT scans (0.625 mm) and preoperative structural T1‐weighted magnetic resonance (MR) images, using the LeadDBS toolbox Version 2.5^16^, ^17^ for DBS electrodes and the Freesurfer package for ECoG.^18^ We excluded sub10 for subdural electrode dislocation.

### 2.3 Experimental protocol and recordings

**FIGURE 1:**
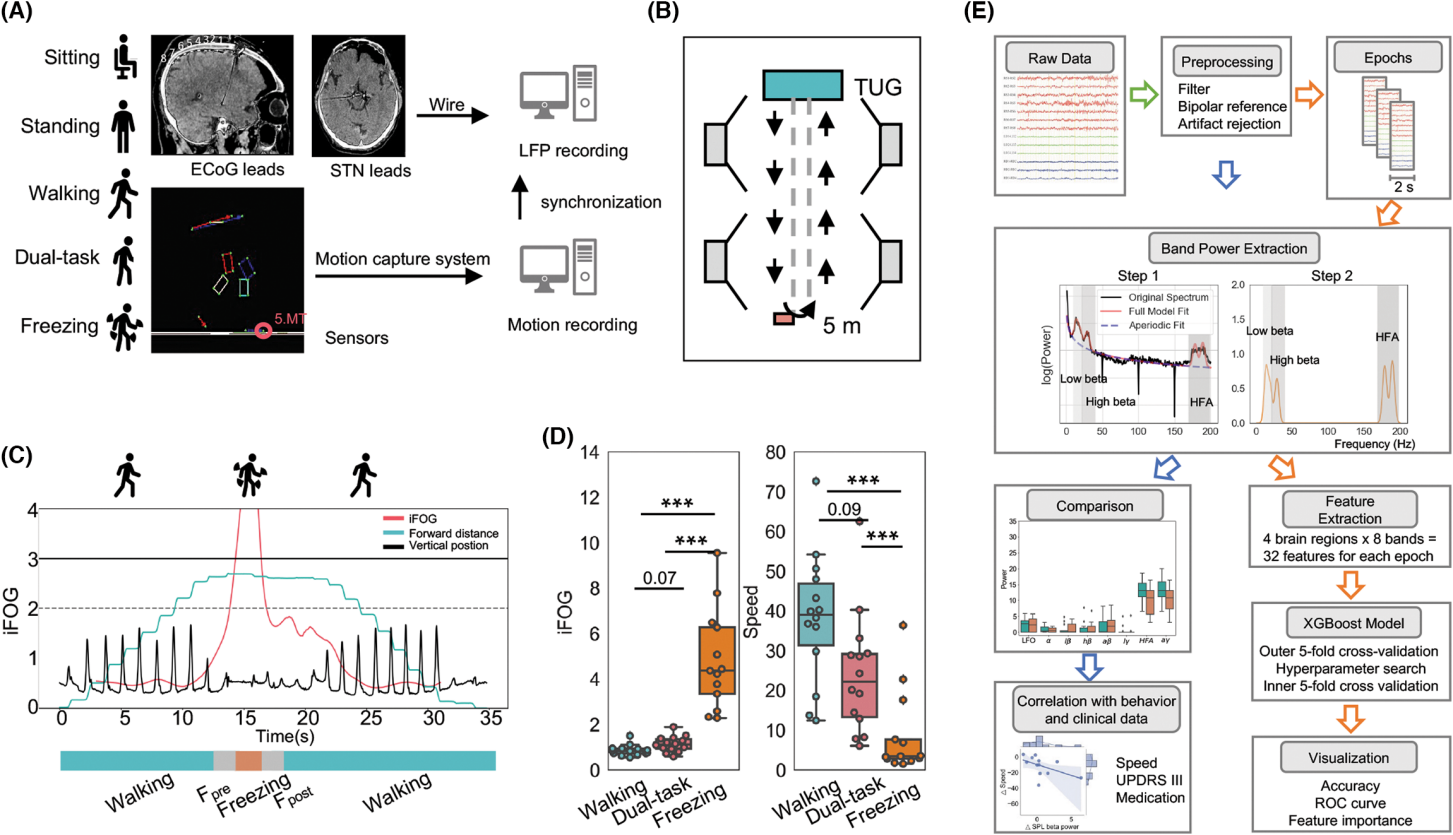
Electrode localization, experimental setup, and analysis pipeline. (A) Subjects undergoing electrocorticogram (ECoG) with bilateral STN lead implantation were studied in five motor statuses (sitting, standing, walking, dual‐task, and freezing). While walking, subjects wore 22 sensors that captured motion. LFP and motion recordings were synchronized. Eight‐contact ECoG electrode localization was visualized in a single patient using fused CT and MRI. Bilateral STN leads were visualized using CT. (B) Walking path. Subjects were asked to complete a 5‐m back‐and‐forth timed up‐and‐go task (TUG) with 3D positions of key body joints captured by an optoelectronic system (four cameras). (C) Illustration of iFOG (freezing index) in a trial. Black lines indicate the vertical positions of the left fifth metatarsal (5.MT), green lines indicate the forward position of the foot, and red lines indicate the iFOG. Freezing was defined as an iFOG >3. We defined 1.5 s before Freezing as “F_pre_,” 1.5 s after F as “F_post_,” and all the other periods as “Walking.” (D) Box plots comparing iFOG and speed between walking, dual‐task walking, and freezing. iFOG of dual‐task and freezing were significantly higher than walking. Speed of dual‐task and freezing were significantly slower than walking (Friedman test for repeated measurements, *p* < 0.001, with a post hoc pairwise Conover's test, ***p < 0.001). (E) Schematic flow chart of preprocessing, band power extraction, power comparison, correlation with behavior and clinical data, feature extraction, machine learning evaluation, and visualization analysis pipeline.

The experiment began in the Gait Laboratory 3–5 days after the electrode implantation. A schematic diagram of the experimental setup is shown in Figure 1. The motor tasks were conducted in the medication “off” (Moff) and medication “on” (Mon) states, with the order randomized across patients (sub02, sub03, and sub05 had only Moff, Table 1). To begin, subjects were asked to sit and stand for at least 3 minutes. During sitting or standing, they remained relaxed and stared at a cross‐sign fixed 2 m immediately ahead of them. Then, the subjects wore motion sensors on key landmark joints of the legs and trunk (Figure 1A), and completed a 5‐m back‐and‐forth timed up‐and‐go task (each counted as one trial). An optoelectronic system (100/200 Hz) was used to measure the 3D positions of the key body joints (CODA, Charnwood Dynamics Ltd, UK). During each trial, patients were instructed to walk at a comfortable speed (walking) or perform additional cognitive tasks while walking (dual‐task). Cognitive tasks were randomly assigned, including calculation, listing animal names, and transferring coins between hands. In both medication states, patients completed at least four trials of walking and performing dual tasks. A synchronized digital video system was used to record the entire experiment.

STN LFP and ECoG recordings were obtained using the JE‐212 amplifier (Nihon Kohden) with a common average and sampling rate of 2000 Hz. Synchronization with additional devices was performed using the “trigger” input to the amplifier.

### 2.4 Walking analysis

Instantaneous speed was calculated as the difference in the walking distance at each adjacent time point (waist channel on each side). We then averaged the instantaneous speed altogether for all time points as the average speed. We adopted a freezing index (iFOG) approach^14^, ^19^ to quantify freezing from the sensor channels' optoelectronic data that were least contaminated (four on each side, including foot, shank, thigh, and waist) (https://github.com/zixiao‐yin/ecogFog). Briefly, iFOG was defined as the ratio of power between the “freezing band” (3–8 Hz) and the “locomotion band” (0–3 Hz) in a 6 s sliding window centered in t with 0.1 s step size. We defined the period whose iFOG was above 3 as “Freeze,” 1.5 s before F as “F_pre_,” 1.5 s after F as “F_post_,” and all the other periods were defined as “Walking” (Figure 1C). Two independent raters evaluated the walking statuses among all the trials and discarded those that were inconsistent.

### 2.5 Local field potential analysis

**FIGURE 2:**
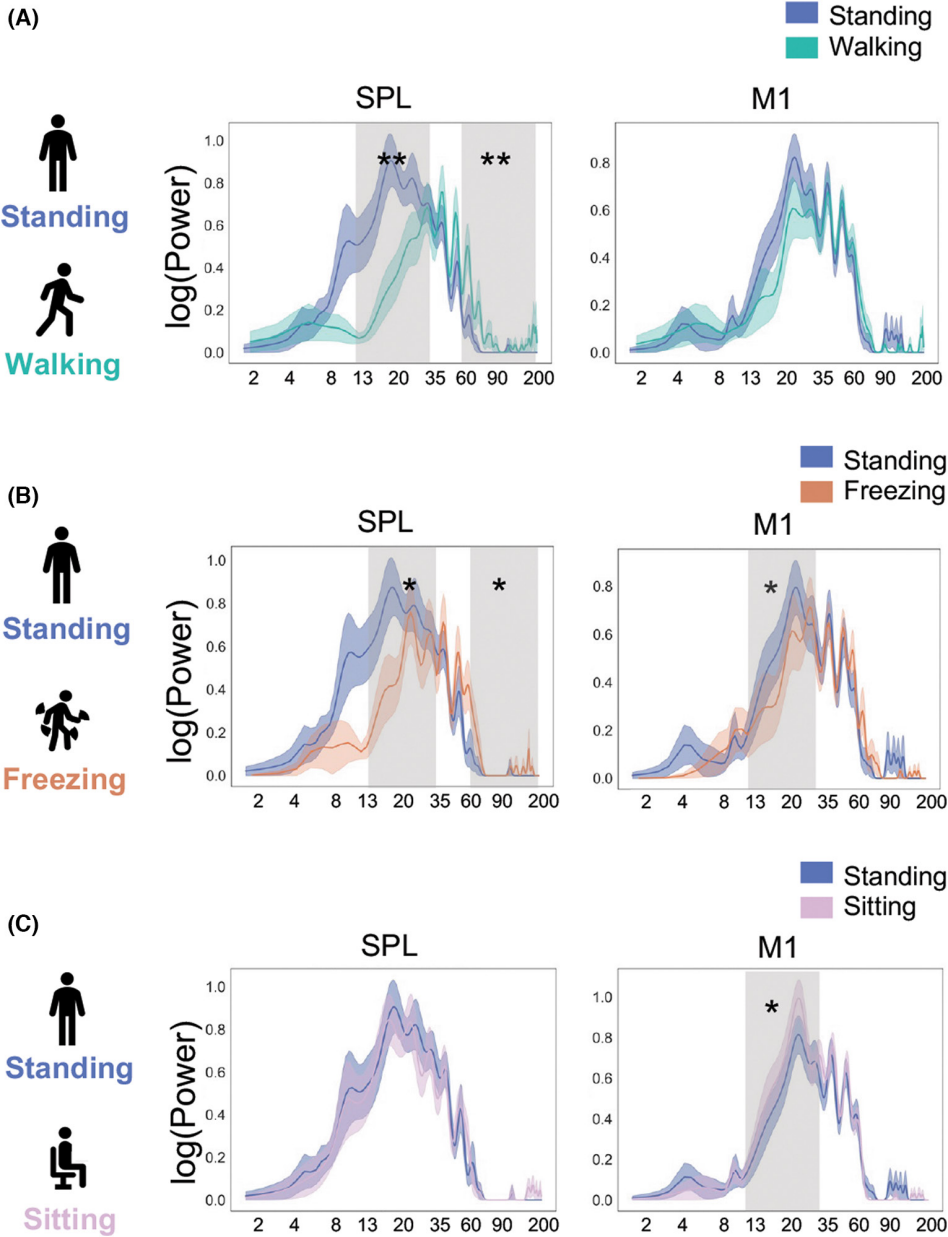
Power differences between motor statuses. (A) Beta and gamma power in the SPL differ between standing and walking status. In the SPL, the beta band decreased significantly from standing to walking (low beta and all beta, *W* = 1.0, *p* = 0.0078; high beta, *W* = 3.0, *p* = 0.019; Wilcoxon signed‐rank test with Bonferroni correction). Gamma power increased significantly from standing to walking (low gamma, *W* = 1.0, *p* = 0.035; high gamma, *W* = 5.0, *p* = 0.039; all gamma, *W* = 0.0, *p* = 0.0039). No significant power difference between standing and walking states was observed in the M1. (B) Beta and gamma power in the SPL and beta power in M1 differ between standing and freezing status. In the SPL, we found that beta power decreased significantly, while all gamma powers increased significantly from standing to freezing (low beta, high beta, all beta, and all gamma, *W* = 0.0, *p* = 0.016). In the M1, beta power decreased significantly when freezing (low beta, W = 1.0, *p* = 0.0039; high beta, W = 8.0, *p* = 0.049; beta, *W* = 6.0, *p* = 0.027). (C) The beta band in the M1 differs between the sitting and standing statuses. The beta power in the M1 decreased significantly from sitting to standing (beta, *W* = 16.0, *p* = 0.041; Wilcoxon signed‐rank test with Bonferroni correction). No significant power difference was observed between sitting and standing status in the SPL.

Electrophysiological recordings were analyzed offline using custom‐written Python scripts based on the MNE package.^20^ A 50 Hz line noise and harmonics were notched out, then band‐pass filtered between 2 and 200 Hz. To improve the spatial specificity, a bipolar montage between adjacent contacts was applied. Trials with obvious artifacts or drift were discarded (3% of trials). To better explore the periodic electrophysiological properties, the LFO, alpha, beta, and gamma bands were identified using an unbiased algorithm that parameterized neural power spectra into periodic and aperiodic components (1/f) (Figure 2E, step 1) to overcome the limitation that standard analytic approaches can conflate periodic parameters with aperiodic ones, and thus, compromising physiological interpretations.^21^ We employed the Welch periodogram method to calculate power spectral density (PSD) (n_overlap = 1000, n_fft = 2000). We subtracted the full model fit by the aperiodic fit to keep the periodic components and calculated the area under the curve (2–8 Hz for low‐frequency oscillations [LFO], 8–13 Hz for alpha band, 13–20 Hz for low‐beta band, 20–35 Hz for high‐beta band, 13–35 Hz for all‐beta band, 60–90 Hz for low‐gamma band, 90–200 Hz for high‐gamma band, and 60–200 Hz for all‐gamma band) in the four regions (Figure 2E, step 2). Gait‐related artifacts were determined by the differences in aperiodic components of the PSD between resting (sitting/standing) and walking. If the walking trial induced an increase in 1/f power bigger than 50% compared to resting, the trial was discarded (2% of the total number of trials).

### 2.6 Machine learning analysis

We performed machine learning analysis to determine which frequency in which region could primarily account for motor status switches, and attempted to classify the patient's motor status. We defined the epoch range of 2 s for the Sitting/Standing/Freezing/Walking/Dual‐task statuses of each subject and calculated the periodic power of LFO, alpha, low‐beta, high‐beta, all‐beta, low‐gamma, high‐gamma, and all‐gamma bands in the PMC, M1, SPL, and STN, resulting in 32 features for each epoch. Missing data were handled by the mean strategy in sklearn SimpleImputer. A nested five‐fold cross‐validation was implemented (Figure S2). The data were divided into 80% for the training set and 20% for the test set. For the imbalanced data, we employed RandomUnderSampler in imblearn by randomly picking samples in all classes except the minority class. Then, we employed a supervised Extreme Gradient Boosting (XGBoost) classifier, which is an ensemble of gradient‐boosted decision trees to predict the motor status.^22^ The cross‐validation inner loop used the training subsets from the outer loop to tune hyperparameters. The random search was applied for hyperparameter optimization and conducted for 25 rounds using the training set only. The hyperparameters are shown in Table S1. A five‐fold cross‐validation scheme was used to evaluate the global classification accuracy (mean ± SD). In addition, we analyzed the receiver–operator characteristic (ROC) curve, confusion matrix, and feature importance of the classifier. For feature importance, we chose default normalized gain values in XGBoost (F score), reflecting the feature's power of grouping similar instances into a more homogeneous child node at the split.^23^

### 2.7 PSD features' correlation with clinical scores and walking speed

We extracted the power of eight‐frequency bands in four regions for each subject during resting statuses (sitting and standing) and examined their Spearman correlation with clinical scales, including UPDRSIII_MOff, MOff_Rigid, and MOff_Tremor. In addition, we calculated the walking speed and task‐induced speed change (△Speed_Task_ = Speed_Dual‐task_ ‐Speed_Walking_), then correlated them with the power change of eight‐frequency bands in four regions for each subject. Furthermore, the correlation of medication‐induced speed change (△Speed_Walking_M_ = Speed_WalkingMOn_ ‐Speed_WalkingMOff_, △Speed_Dual‐task_M_ = Speed_Dual‐taskMOn_ ‐Speed_Dual‐taskMOff_) with SPL beta power change was also calculated. A linear mixed‐effect model was used for repeated measures data, where the dependent variable was speed, the fixed effects were beta power and medication state, and the random effect was the subject.

### 2.8 Statistics

The normality of the data was examined using the Shapiro–Wilk test. The data were presented as the median (range) due to skewed distribution. The Friedman test with post hoc pairwise Conover's test was used to compare the iFOG and speed among walking, dual‐task, and freezing. The Wilcoxon signed‐rank test was used to investigate the power difference in eight‐frequency bands, with Bonferroni correction for multiple comparisons. A two‐tailed *p*‐value <0.05 was considered significant. All statistical analyses were performed in Python 3.

## 3 RESULTS

After quality inspection, a total of 325 trials were further analyzed. In the medication “off” state, we obtained 3914.3 s of sitting, 3940.6 s of standing, 1069.5 s of walking, 2031.4 s of dual‐task, and 1758.4 s of freezing. For the medication “on” state, we acquired 3642.8 s of sitting, 2544.0 s of standing, 656.3 s of walking, 1515.0 s of dual‐task, and 54.0 s of freezing.

### 3.1 Beta and gamma power in the SPL differ between standing and walking statuses

In the SPL, we found that the beta band decreased significantly from standing to walking (low beta and all beta, *p* = 0.0078; high beta, *p* = 0.019). In addition, the gamma power increased significantly from standing to walking (low‐gamma, *p* = 0.035; high‐gamma, *p* = 0.039; all gamma, *p* = 0.0039). Nevertheless, no significant difference in power between the standing and walking statuses was observed in the M1, PMC, or STN (Figure 2A).

### 3.2 Beta and gamma power in the SPL and beta power in the M1 differ between standing and freezing statuses

When freezing occurs during walking, the patient's feet cease to move and the walking speed is slow, which is similar to a voluntary stop, whose corresponding status in this study was standing. Then, we compared the power between freezing and standing among the 12 subjects who exhibited a freezing gait while walking. In the SPL, we found that the beta power decreased significantly, while all gamma powers increased significantly from standing to freezing (low beta, high beta, all beta, and all gamma, *p* = 0.016). In the M1, the beta power decreased significantly when freezing (low beta, *p* = 0.0039; high beta, *p* = 0.049; beta, *p* = 0.027). No significant differences were observed in the PMC or STN (Figure 2B).

### 3.3 Beta power in the M1 differs between sitting and standing status

We found that the beta power in the M1 decreased significantly from sitting to standing (beta, *p* = 0.041). No significant power difference was observed between sitting and standing statuses in the SPL, PMC, or STN (Figure 2C).

### 3.4 The beta power in the SPL ranks the highest at differentiating the five statuses and correlates with speed in the medication “off” state

**FIGURE 3:**
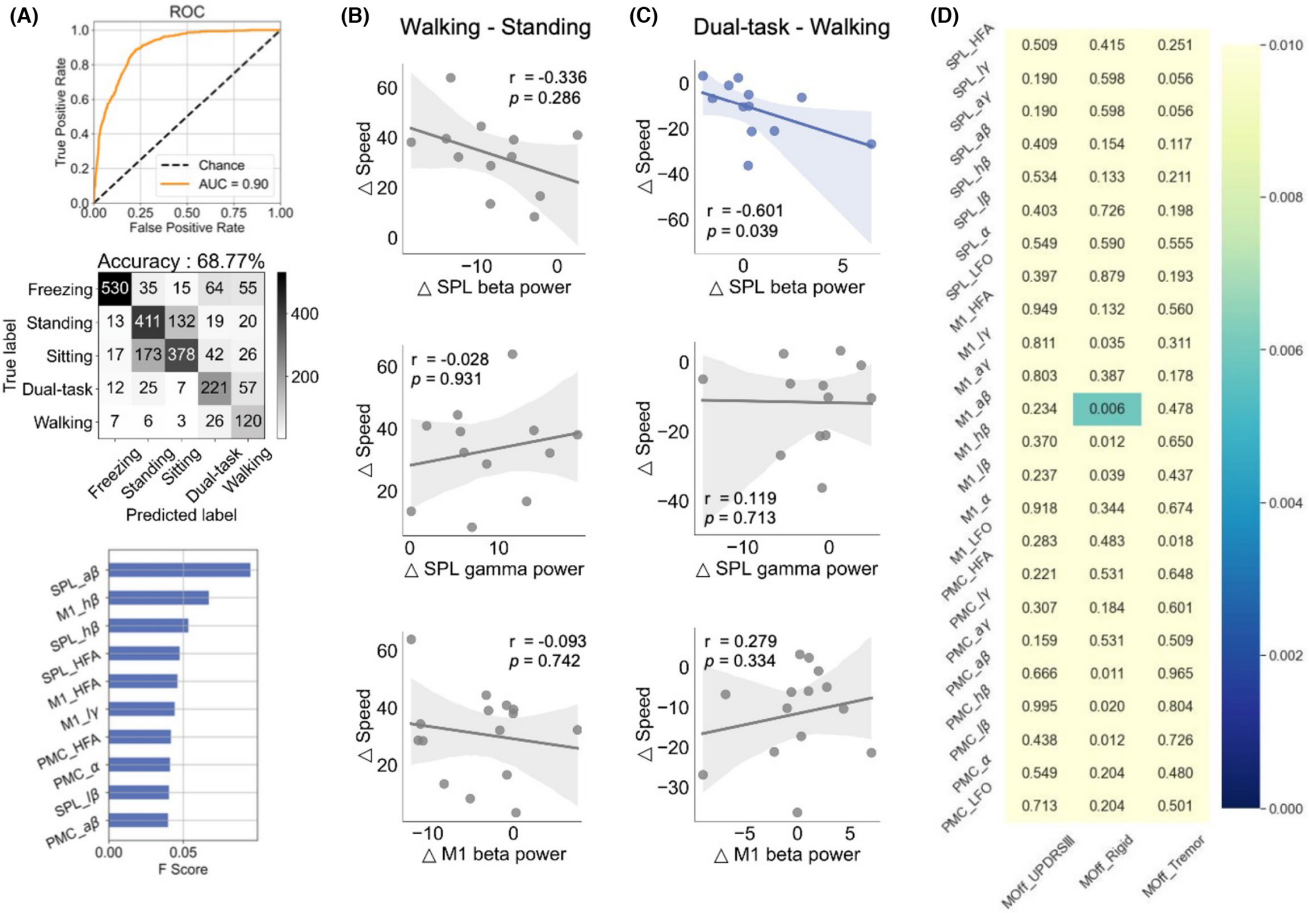
Beta power in the SPL was the best at differentiating the five statuses and correlated with speed in the medication “off” state. (A) The machine learning XGBoost algorithm classified the five statuses (freezing/standing/sitting/dual‐task/walking) with an AUC of 0.90 and an accuracy of 68.77% across participants. Beta power in the SPL ranked highest in feature importance. (B) No significant correlations were found between beta and gamma power in the SPL or beta in the M1 and walking speed. (C) Task‐induced increases in SPL beta power were negatively correlated with reductions in speed (dual‐task–walking, *r* = −0.601, *p* = 0.039). No correlations were found between gamma power in the SPL or beta in the M1 and speed. (D) Exploration of power correlation with clinical scores during standing. Significant correlations were only observed between beta power in the M1 and the rigidity score (*p* = 0.006 < 0.05/8). No significant correlations were detected in the SPL or PMC.

The XGBoost classification algorithm was employed to differentiate the five statuses (freezing/standing/sitting/dual‐task/walking) in the medication “off” state. The classifier had accuracies of 68.77% in cross‐validation performance with an AUC of 0.90. In addition, the beta band in the SPL ranked highest in feature importance (Figure 3A).

As we observed that the beta and gamma bands in the SPL, and the beta bands in the M1 were significantly different between statuses, we aimed to explore the role of such oscillations. First, we did not observe a correlation between the power of the bands and walking speed at the group level (Figure 3B). A task‐induced increase in the SPL beta power was significantly negatively correlated with a decrease in speed (dual‐task–walking, *n* = 12) (Figure 3C). No correlations were observed between the gamma power in the SPL or the beta power in M1 and speed.

In addition, we correlated eight bands in the SPL, M1, and PMC when sitting or standing with clinical scores (UPDRS III, rigidity, and tremor scores). Significant correlations were only observed between the beta power in the M1 and the rigidity score while standing (*p* = 0.006 < 0.05/8). No significant correlations were detected in the SPL or PMC while standing (Figure 3D), and no significant correlations between power and clinical scores were found while sitting.

### 3.5 Beta power in the SPL ranks highest in differentiating the five statuses in medication “on,” and correlates with the walking speed changes induced by medication

**FIGURE 4:**
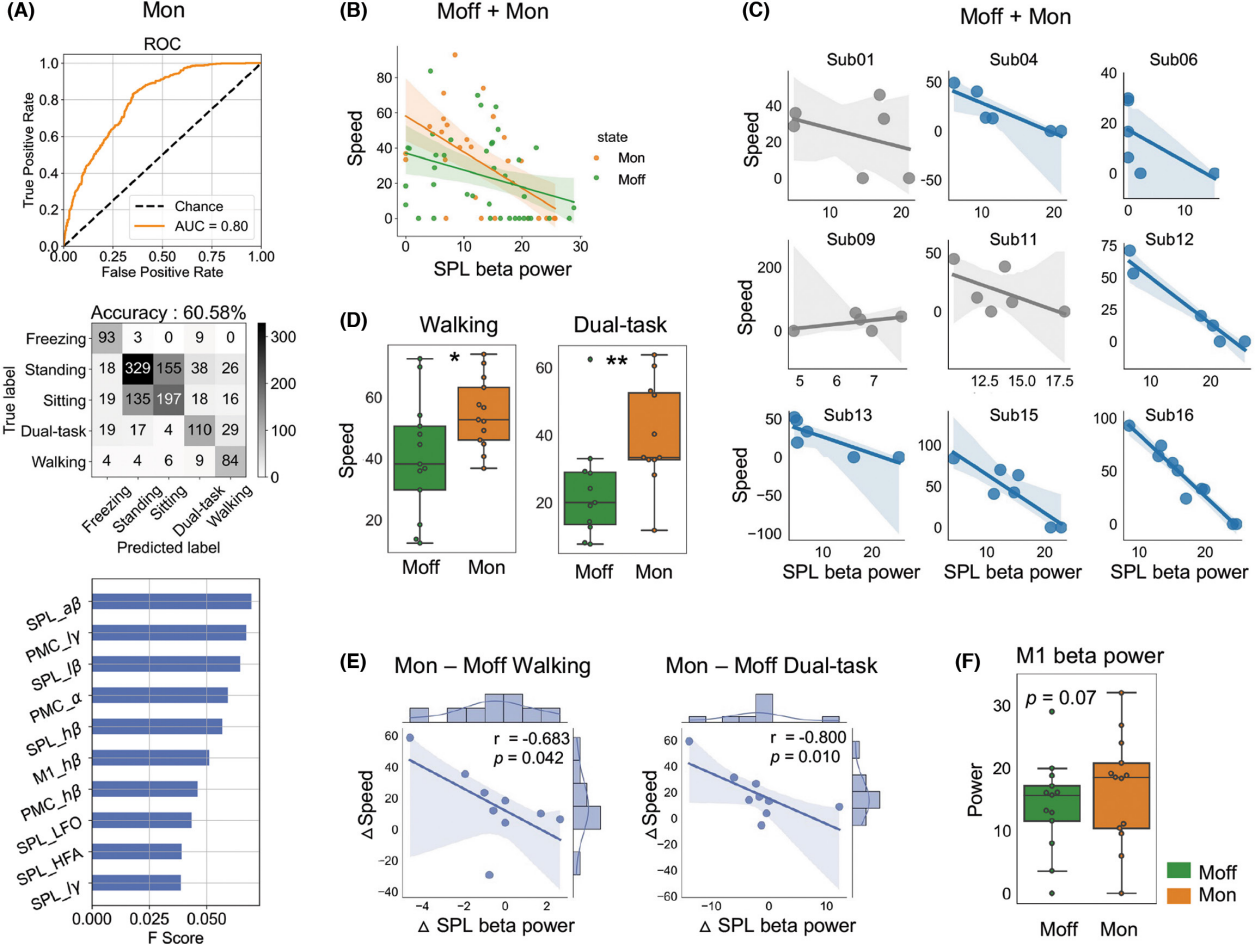
Beta power in the SPL ranks highest in differentiating the five statuses and correlates with speed in the medication “on” state. (A) The machine learning XGBoost algorithm classified the five statuses (freezing/standing/sitting/dual‐task/walking) with an AUC of 0.80 and an accuracy of 60.58% across participants in the medication “on” state. Beta power in the SPL ranked highest in feature importance. (B) Significant correlations of SPL beta power and speed were observed in both the medication “off” and “on” states in linear mixed‐effect model (*p* = 0.000). (C) The correlations of SPL beta power and speed at the subject level, after combining the medication “off” and “on” states. Significant negative linear correlations were detected in six of the nine subjects (significant correlations were plotted in blue and non‐significant correlations were in gray). In sub16, a total of 10 walking statuses were examined and good correlations were observed (Spearman *r* = −0.948, *p* = 0.000). (D) Box plots comparing the average speed between the medication “on” and “off” states while walking or dual‐task walking. Walking speed in the medication “on” state was significantly faster than that in the medication “off” state (*p* = 0.026, *n* = 13, Wilcoxon signed‐rank test). Dual‐task walking speed in the medication “on” state was significantly faster than that in the medication “off” state (*p* = 0.0068, *n* = 11, Wilcoxon signed‐rank test). (E) Significant correlation of medication‐induced SPL beta power changes with speed changes when walking (Spearman *r* = −0.683, *p* = 0.042) and dual‐task (Spearman *r* = −0.800, *p* = 0.010) at the group level. (F) No significant medication‐induced changes were observed in M1 beta power while standing.

Next, we explored whether the electrophysiological characteristics above remained true in the medication “on” state. First, the XGBoost algorithm reached an AUC of 0.80 and a mean accuracy of 60.58%. The beta band in the SPL also ranked highest in feature importance. In addition, low gamma and alpha bands in the PMC ranked relatively high in feature importance (Figure 4A).

Then, we combined the data for all patients in both the medication “off” and “on” states, and observed significant correlations between SPL beta power and speed (linear mixed‐effect model, *p* = 0.000) (Figure 4B). We also examined the correlations between beta power in the SPL with speed at the subject level, combining the medication “off” and “on” states (Figure 4C). Significant negative linear correlations were detected in six of nine subjects. In sub16, a total of 10 walking statuses were examined and good correlations were observed (Spearman *r* = −0.948, *p* = 0.000).

We found that the subjects walked significantly faster in walking and dual‐task walking after taking medication. In addition, medication significantly reduced the iFOG during dual‐task walking (Figure 4D). We tested the correlation between medication‐induced SPL beta power changes and speed changes during walking and dual‐task walking. Significant negative linear correlations were both detected at the group level (Figure 4E).

For M1 beta power, no significant medication‐induced power changes were observed when resting (standing shown in Figure 4F). No significant power correlations with clinical scores were found in the M1 during standing in the medication “on” state.

## 4 DISCUSSION

We recorded the intracranial neurophysiology of PD patients while sitting, standing, walking, dual‐task walking, and freezing and detected significant power differences in the SPL and M1. First, in the SPL, the beta and gamma bands played essential roles in walking status switches. Second, the SPL beta power was negatively correlated with walking speed, and the beta power changes were also negatively correlated with walking speed changes induced by the task or medication. Third, beta power in M1 could reflect the degree of rigidity in the medication “off” state and could be reversed by medication. Finally, XGBoost algorithms were employed to distinguish five motor statuses with a mean accuracy of 68.77% in the medication “off” state and 60.58% in the medication “on” state.

### 4.1 SPL beta band in motor status switching and its negative linear correlation with walking speed

The SPL provides a bridge for sensorimotor transformations in motor, including motor awareness and action planning.^24^, ^25^, ^26^ The SPL has been implicated in goal‐directed movements and is thought to be extremely important in integrating motor plans with information from stimuli in the visual environment.^27^, ^28^ In this study, we detected significant differences in SPL beta and gamma power between standing and walking, and standing and freezing in pairwise comparisons. We found that the feature importance of the SPL beta power ranked highest in distinguishing among walking, dual‐task, and freezing statuses. Based on our results of the machine learning algorithm to classify the five motor statuses, the importance of SPL beta power also outweighed other features. Previous studies showed that beta oscillations were prevalent throughout the motor system and were modulated during reach tasks by the right parietal cortex, which was a crucial hub for movement execution.^29^ Regarding the gamma band in the SPL, our results supported that its synchronization encodes motor goals during visuomotor processing for saccades.^30^, ^31^

We also detected a negative linear correlation of SPL beta power with speed. First, in the medication “off” state, the change in SPL beta induced by task was negatively correlated with a reduction in speed at the group level. Nevertheless, we did not find a significant correlation between the decrease in SPL beta induced by walking with walking speed (*p* = 0.286), partly due to the small number of subjects. Second, in both the medication “off” and “on” states and at the group level, significant correlations of SPL beta power and speed were observed. Third, at the single‐subject level, beta power in the SPL was mainly linearly correlated with walking speed in the “Moff + Mon” states. Fourth, medication‐induced changes in SPL beta power were also negatively correlated with changes in speed, both during walking and dual‐task statuses. A previous EEG study observed walking speed‐related desynchronization in SPL beta in healthy elderly individuals.^32^ SPL beta desynchronization is considered an electrophysiological correlate of increased cortical activation for movement production and/or the processing of sensory information.^33^ Our study verified the linear correlation between beta power in the SPL with walking speed. Thus, we consider SPL beta as a relatively stable electrophysiological biomarker for walking speed.

### 4.2 M1 beta power and rigidity

In this study, we found that the beta power decreased from sitting to standing and from standing to freezing. Studies of M1 spectral power in non‐human primates during natural locomotor behavior revealed that beta event‐related desynchronization (ERD) was modulated by gait, thus, illustrating its contribution to movement sequence and coordination.^34^, ^35^ In humans, beta oscillations captured by EEG channels near the M1 have been considered as biomarkers for motor activity, whose power will decrease from a non‐moving baseline to walking.^36^ In addition, the changes in power between sitting and standing underline M1 beta band's role in postural maintenance.^37^, ^38^ Moreover, we detected a negative correlation between M1 beta and rigid score under medication “off” state when standing. These findings are consistent with previous studies in non‐human primates that found that M1 beta power decreased with the progression of the disease (especially with worsening of bradykinesia),^39^ and a human magnetoencephalography (MEG) study that found that M1 beta power was inversely correlated with rigidity scores.^36^

### 4.3 Medication‐induced changes in key electrophysiological biomarkers

Several studies have explored the effects of levodopa on the cortical beta in PD patients using EEG and MEG.^40^, ^41^ An increase in beta power after taking the medication was observed to be correlated with an improvement in rigidity and bradykinesia.^36^, ^42^, ^43^ In the current study, no significant M1 beta power changes were observed after taking the medication, despite that there was an increasing trend (*p* = 0.07). Beta in the M1 ranked high in feature importance and correlated with rigidity in the medication “off” state. Medication tended to increase the beta power in the M1 and decreased its importance in status switching. In addition, no significant correlations of M1 beta power with rigidity were found in the medication “on” state. Thus, we speculate that M1 beta band is a pathological activity that reflects the degree of rigidity, and could be reversed by medication. Notably, beta in the SPL ranked highest in feature importance and maintained a relatively good linear correlation with speed in the medication “on” and “off” states; thus, we consider it a stable physiological indicator of walking activity. For feature importance ranking, M1 and PMC ranked second in Moff and Mon, respectively. Hence, we consider that the key electrophysiological biomarkers are different between the medication “on” and “off” states.

### 4.4 Application of the XGBoost algorithm in human intracranial signals for movement decoding

Machine learning (ML) algorithms can be trained to make classifications or predictions and to uncover critical insights in data mining. To date, only a few investigations implementing ML methods to decode human pathological or physiological states with intracranial signals, and in adaptive DBS, have been published, with accuracies of 0.55–0.96.^44^ In the current study, we employed the XGBoost classifier because it yields improved model performance and parallelized computational speeds compared to other decision tree‐based models. Previous studies demonstrated that it performs best among many models for ECoG movement decoding^45^, ^46^ and EEG emotional neural‐state identification.^47^ In this study, five‐class classification accuracy reached an average of 68.77% in Moff and 60.58% in Mon states. In addition, the algorithm revealed that SPL beta power had the greatest importance in differentiating motor statuses. Hence, we applaud XGBoost because of its advantages of effectively integrating multivariate features, which may further augment the capabilities of functional and dysfunctional statuses encoding.

### 4.5 Limitations

The present study suffered from several limitations. First, we had a relatively small amount of data; some ECoG electrodes did not cover the SPL, incomplete statuses were observed for several subjects, and movement‐induced artifacts were also observed. Second, we performed a retrospective analysis to train and evaluate the model but did not perform real‐time decoding. Thus, our future work is to study real‐time signal changes during the motor status switches.

## 5 CONCLUSION

Our results showed that the beta bands in the SPL indicated the physiological walking activities both in the medication “off” and “on” states and beta power in M1 reflected the degree of rigidity and could be reserved by medication. Moreover, the XGBoost algorithm could effectively classify different motor statuses in PD patients. Our study provides evidence for decoding PD patients' movements and sheds new insight into the development of intelligent closed‐loop DBS.

## AUTHOR CONTRIBUTIONS

J.Z., Y.J., Q.Z., and B.Z. designed the study; Q.Z., H.X., B.Z., Z.Y., Y.L., D.L., Y.B., G.Z., G.Q., L.S., A.Y., and F.M. acquired and analyzed the data; Q.Z. and Y.J. contributed to drafting the text and preparing the figures; Q.Z., H.X., B.Z., Z.Y., Y.G., R.T., Y.J., and J.Z. reviewed and revised the manuscript.

## CONFLICT OF INTEREST STATEMENT

None.

## Supporting information


Figures S1–S2.

Table S1.

## Data Availability

The data that support the findings of this study are available from the corresponding author upon reasonable request.
