# Supplementary material for: Fronto‐parieto‐subthalamic activity decodes motor status in Parkinson's disease
Source: CNS Neurosci Ther. 2023 Apr 5;29(7):1999–2009. doi: 10.1111/cns.14155 (PMC10324359; doi:10.1111/cns.14155)
Supplement: Supplementary file 1 — Figures S1–S2. [file CNS-29-1999-s002.docx]

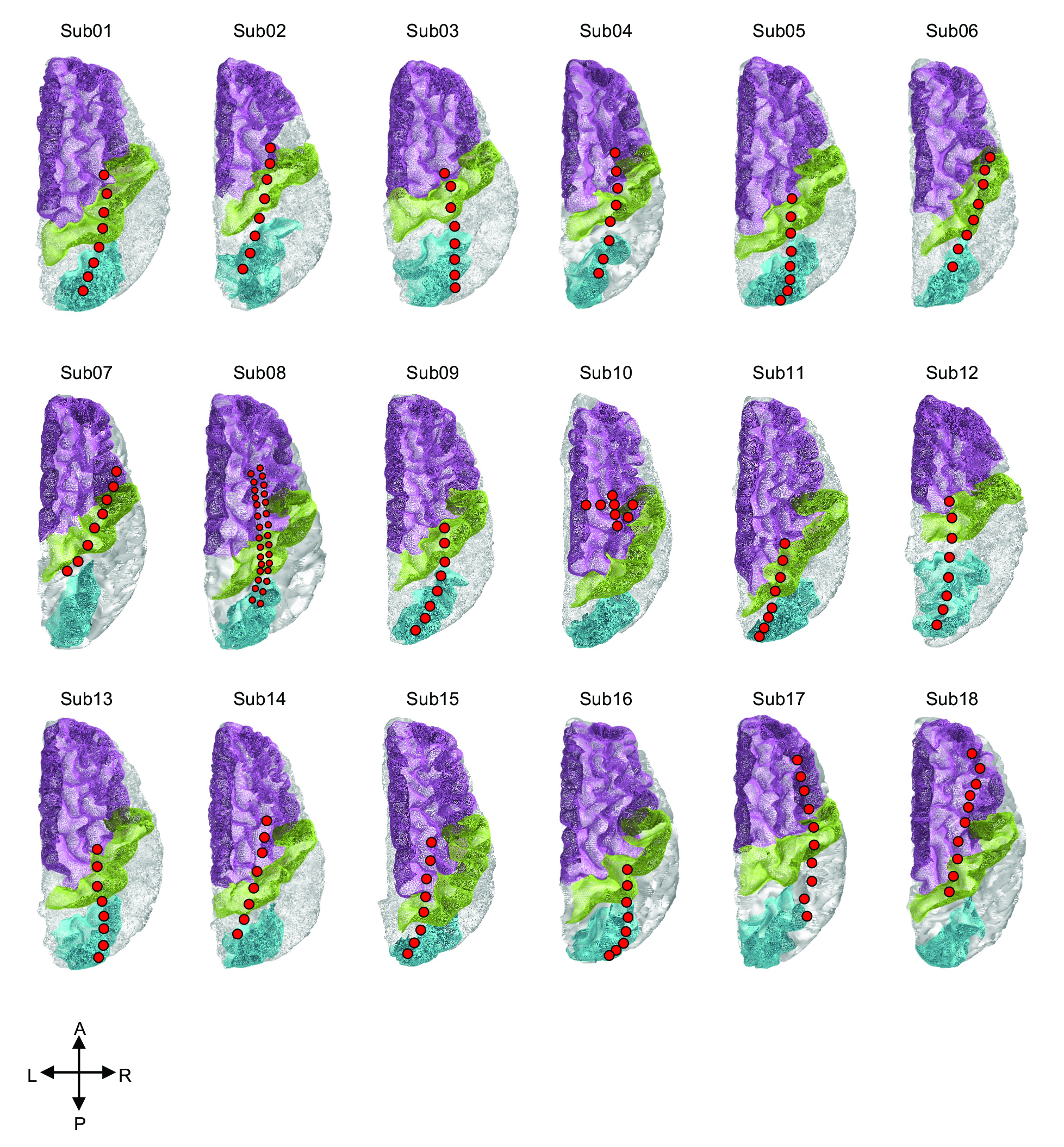


**Figure S1.** Subdural electrode localization for each subject.

The subdural strip electrodes were placed in the right hemisphere. Red dots represent the ECoG electrodes. The eight-contact subdural strip electrodes (two subjects with 10-contact strip electrodes, and one subject with 30-contact strip electrodes arranged in two rows) covered the PMC (posterior part of the pink region), M1 (highlighted in green), S1, and SPL (highlighted in blue). A: anterior, P: posterior, L: left, R: right.


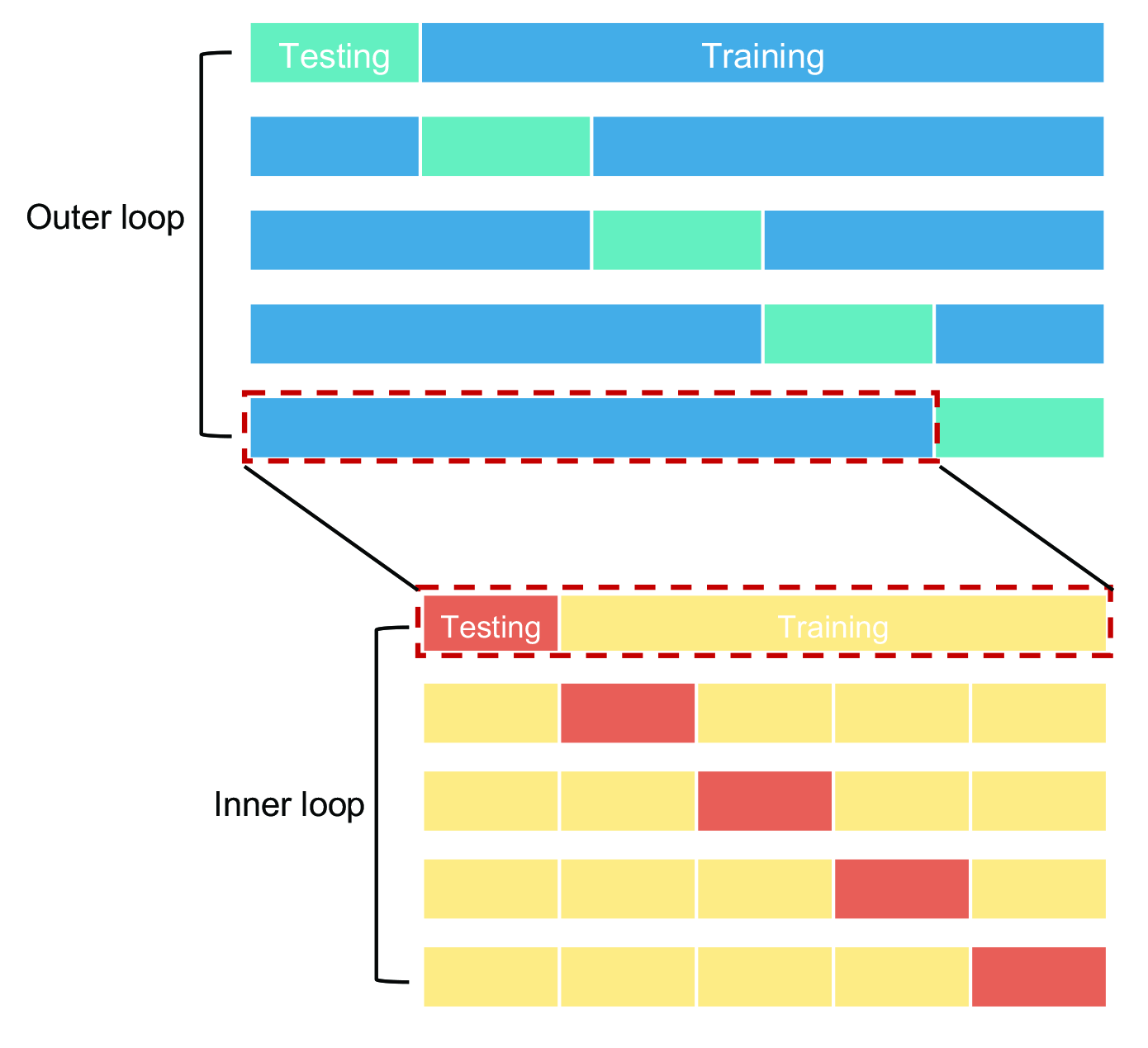


**Figure S2.** Illustration of the 5-fold validation test set.

The full dataset was split and stratified into five folds. Each fold was iteratively used as a test fold and the rest was used for training. The cross-validation inner loop used the training subsets from the outer loop to tune hyperparameters.
