# Supplementary material for: Fronto‐parieto‐subthalamic activity decodes motor status in Parkinson's disease
Source: CNS Neurosci Ther. 2023 Apr 5;29(7):1999–2009. doi: 10.1111/cns.14155 (PMC10324359; doi:10.1111/cns.14155)
Supplement: Supplementary file 2 — Table S1. [file CNS-29-1999-s001.docx]

Supplementary Table 1. Hyperparameters of XGBoost

| n_estimators | 50 |
| --- | --- |
| base score | 0.5 |
| ﻿early stopping rounds | 20 |
| eta | 0.3 |
| ﻿gamma | 0 |
| ﻿max depth | 8 |
| ﻿min child weight | 5 |
| ﻿max delta step | 0 |
| ﻿sampling method | uniform |
| ﻿L1 regularization | 0 |
| ﻿L2 regularization lambda | 0.1 |
| ﻿num parallel trees | 1 |
